# Does the use of an electronic reminder device with or without counseling improve adherence to lipid-lowering treatment? The results of a randomized controlled trial

**DOI:** 10.3389/fphar.2013.00069

**Published:** 2013-05-29

**Authors:** M. J. Kooy, B. L. G. van Wijk, E. R. Heerdink, A. de Boer, M. L. Bouvy

**Affiliations:** Division of Pharmacoepidemiology and Clinical Pharmacology, Utrecht Institute for Pharmaceutical Sciences, Utrecht UniversityUtrecht, Netherlands

**Keywords:** medication adherence, pharmacy, electronic reminder device, intervention

## Abstract

**Background:** Lipid-lowering treatment with statins has proven to be effective in reducing cardiovascular events and mortality. In daily practice, however, adherence to medication is often low and this compromises the therapeutic effect. The aim of this study was to assess the effectiveness of an electronic reminder device (ERD) with or without counseling to improve refill adherence and persistence for statin treatment in non-adherent patients.

**Methods:** A multicenter, community pharmacy-based, randomized controlled trial was conducted in 24 pharmacies in the Netherlands among patients with pre-baseline refill adherence rates between 50 and 80%. Eligible patients aged 65 years or older were randomly assigned to 1 of 3 groups: (1) counseling with an ERD (*n* = 134), (2) ERD with a written instruction (*n* = 131), and a (3) control group that received the usual treatment (*n* = 134).

**Main outcome measure:** refill adherence to statin treatment for a 360-day period after inclusion (PDC360). Patients with a refill rate ≥80% were considered adherent. The effect among subgroups was also assessed.

**Results:** There were no relevant differences at baseline. In the counseling with ERD group 54 of 130 eligible patients received the counseling with ERD. In the ERD group, 117 of 123 eligible patients received the ERD. The proportions of adherent patients in the counseling with ERD-group (69.2%) and in the ERD group (72.4%) were not higher than in the control group (64.8%). Among women using statins for secondary prevention, more patients were adherent in the ERD group (86.1%) than in the control group (52.6%) (*p* < 0.005). In men using statins for secondary prevention the ERD was found to have no effect.

**Conclusion:** In this randomized controlled trial, no statistically significant improvement of refill adherence was found if an ERD was used with or without counseling. However, in a subgroup of women using statins for secondary prevention the ERD did improve adherence significantly.

## Introduction

Statins are effective in reducing cardiovascular events and mortality (West of Scotland Study Group, 1997). Despite this beneficial effect, adherence to lipid-lowering treatment is substantially worse in daily practice than in the controlled setting of randomized controlled trials (Jackevicius et al., 2002; Eussen et al., 2010). Non-adherence to statins reduces the beneficial effect and increases the risks of cardiovascular events (Simpson and Mendys, 2010).

Urquhart and Vrijens (2005) and more recently Vrijens et al. (2012) have argued that three phases of chronic drug treatment can be identified: acceptance of the treatment plan, execution of the drug regimen and eventually complete discontinuation (non-persistence) of treatment. Non-adherence can take place in these three different stages (Vrijens et al., 2012). In this study we focused on patients who are non-adherent in the execution phase and defined this as “the extent to which a patient acts in accordance with the prescribed interval and dose of a dosing regimen” (Cramer et al., 2008).

Numerous attempts have been made to design interventions to improve medication adherence for patients with chronic diseases, with variable rates of success (Haynes et al., 2002, 2008; McDonald et al., 2002; Eussen et al., 2010; Schedlbauer et al., 2010). Almost all effective interventions were complex, incorporating combinations of more convenient care, information, reminders, self-monitoring, reinforcement, counseling, family therapy, and other forms of additional supervision or attention by a healthcare provider (Haynes et al., 2008; Schedlbauer et al., 2010). Johnson et al. (2006) examined the application of the full “stages of change model” to increase patients' taking adherence to lipid-lowering treatment and patients who were treated according to this model were more likely to be adherent at 12 and at 18 months after intervention. This model is based on five different stages of change: the pre-contemplation stage, contemplation stage, preparation stage, action stage and maintenance stage (Prochaska et al., 1992). However, an intervention as simple as a medication reminder has also been demonstrated to increase adherence to antihypertensive drugs by about 6–8% (Da Costa et al., 2005; Christensen et al., 2010) and lipid-lowering treatment by 6.5–12% (Vrijens et al., 2006). A medication reminder aims to minimize forgetfulness, which is a common reason for non-adherence (Stone, 2001).

Studies based on pharmacy records suggest that these refill data can be used to identify non-adherent patients (Yang et al., 2003; Chapman et al., 2005; Perreault et al., 2005). Community pharmacists could play an important role in improving adherence (Eussen et al., 2010; Morgado et al., 2011; Taitel et al., 2012). We therefore used refill data to select non-adherent statins users and we designed a pharmacy-based intervention. There are at least two reasons why adherence-improving interventions are not successful: (1) patients who volunteer to participate in the study were already adherent at baseline (Van Wijk et al., 2005; Eussen et al., 2010) and (2) participation in the study can also increase adherence in the control group [the Hawthorne effect (Morris and Lamb, 1989)], thereby decreasing the study's power to detect a significant effect of the intervention. In this study we used a framework that minimizes these two limitations. Firstly, only non-adherent patients were included. Secondly, adherence was assessed objectively with refill data. Finally patients were not aware of study participation, thereby minimizing the Hawthorne effect. The object of this study was to assess the effectiveness of an electronic reminder device (ERD) with or without counseling to improve refill adherence and persistence for statin treatment in non-adherent patients.

## Methods

### Study-design

**Figure 1:**
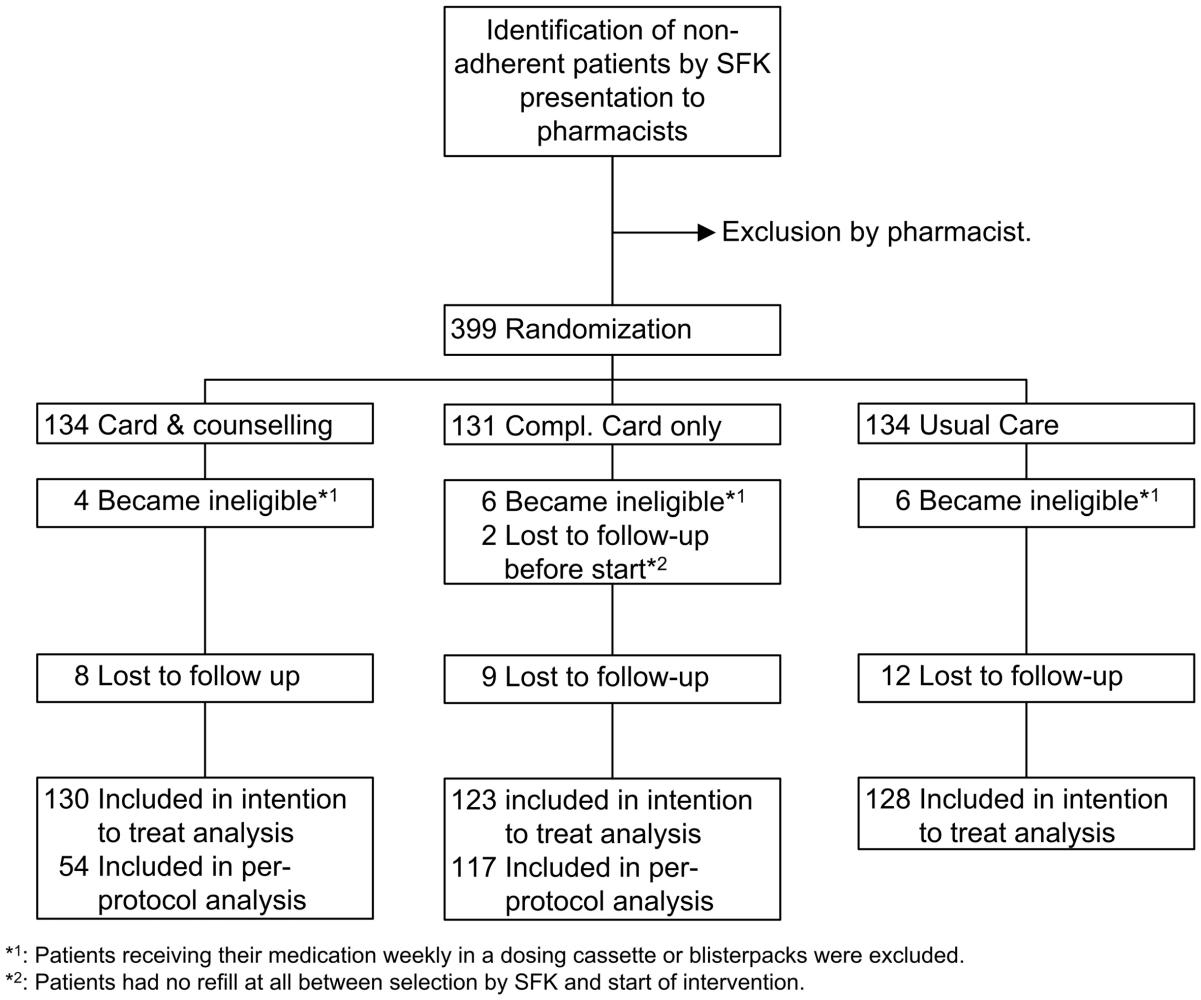
**Trial profile**.

A multicenter, community pharmacy-based, randomized controlled trial (Figure 1).

### Participants

Patients were selected in 24 community pharmacies in different areas of the Netherlands. We included patients who had started statins at least one year prior to inclusion and were non-adherent in the year prior to inclusion (refill rate between 50 and 80%). We excluded patients who were not personally responsible for their medication intake or who received their medication in a dosing aid, patients with a life expectancy of less than 6 months and patients younger than 65 years. Life expectancy is difficult to assess but this assessment was based on personal knowledge about the patient and the prescription of drugs used in the palliative phase. Patients who had switched to a different statin in the 540 days before the inclusion date were also excluded.

General practitioners received general information about the study but were not involved in the recruitment or selection. Patients were not asked to consent to study participation. Patients were recruited between January 2008 and March 2008. *The Medical Ethics Review Committee (METC) of the University Medical Centre Utrecht considered our research proposal in its meeting dated 21 August 2007 and concluded that the Dutch Medical Research Involving Human Subjects Act (WMO) was not applicable (approval number 07-226) and therefore the METC's approval of the study was not needed*. No extra data had to be collected and there was no extra burden for the patients in the usual care group. Patients in the intervention groups could refuse the intervention. The trial was registered at www.clinicaltrials.gov under the identifier NCT00493337.

Pharmacists were informed about the study and received instructions about the randomization and intervention.

### Definition of non-adherence for selecting patients

Patients were selected based on refill data and were presented to the pharmacist when they met the following criteria: (1) received a prescription for a statin in the preceding month, (2) received a prescription for the same statin between 12 and 18 months prior to that prescription and (3) had a refill adherence between 50 and 80% of the 365 days prior to the last statin prescription covered by the same statin (see below). For patients with more than 60 *consecutive* days without coverage, (4) an additional refill of a non-statin prescription was required to exclude the possibility that the patient had moved to another pharmacy. Refill adherence was assessed by calculating the proportion of the 365 days covered before selection by using the dispensing date and the theoretical duration of a prescription. The latter is assessed by dividing the number tablets dispensed by the number of tablets used daily, both available from the pharmacy computer system. In the Netherlands 95% of patients collect their prescription drugs in the same community pharmacy (Buurma et al., 2008).

Patients were identified by an automated search-protocol developed by the “Stichting Farmaceutische Kengetallen” (SFK). The SFK collects dispensing data from more than 90% of Dutch community pharmacies. The results of the selection were presented to the pharmacist on a secure website. The pharmacists were asked to assess if each patient was eligible. After selection by the pharmacist, patients were randomized in to 1 of the 3 intervention groups.

The 80% cut-off value is the most frequently used value for non-adherence although its clinical relevance depends on the particular medication under study (Andrade et al., 2006). Karve et al. (2009) found that among patients treated for hyperlipidemia, a cut-off value of 81% was clinically relevant with regard to diseased-related hospitalization. Patients with an adherence of less than 50% were excluded to increase the likelihood that patients were suboptimal users rather than complete discontinuers who restarted treatment.

### Randomization

Patients were randomized into one of three groups: the Counseling with ERD group, the ERD group (with written instruction) or the control group (usual care) in a 1:1:1 ratio using a computer generated random number sequence. Patients were randomized in blocks based on baseline medication adherence (above or below 65%) and age [above or below 75 using the minimization method with equal weights assigned to both categories (Scott et al., 2002; Heritier et al., 2005)].

### Intervention

#### Counseling with ERD group (1)

The pharmacist sent patients a written invitation and a followup phone call was made 14 days after the written invitation (see Appendix 1). The intervention consisted of two elements: the first and most important element was the application of the stages of change model in non-adherence counseling. The second element was the ERD.

**Figure 2:**
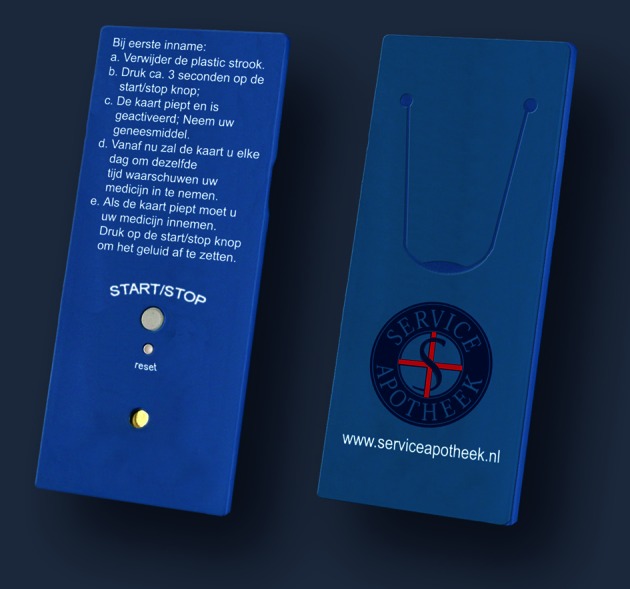
**ERD, Compliance Card®.** This credit-card size ERD needs to be activated after the first dose and gives a signal after every 24-h interval following its activation. It actively needs to be turned off. An instruction for the first use is printed on the card.

The 10-min counseling session by the pharmacist consisted of five phases. The patient received feedback on their previous drug dispensing data (1). Patients were asked if they were aware that they were non-adherent and reasons for non-adherence were discussed (2). Patients were informed about the benefits of statin use (3), received an ERD to help them with medication taking (4) and were informed that after one year they would be invited for a follow-up visit (5). The ERD (Compliance Card®, Figure 2) is a medication reminder device that starts beeping every day at the same time until the patient switches it off. Patients can adjust the time.

#### ERD group (2)

Patients received the ERD by mail with a written instruction about the use of the device (see Appendix 2).

#### Control group (3)

Patients in the control group received usual care. In the Netherlands usual care entails: at the start of therapy, patients receive written and spoken information about the therapy and medication. After about 2 weeks, the patient should return for the first refill. The patient is then asked about his or her experience, concerns and need for information. Patients who use a statin for more than a year do not receive counseling on a regular basis.

### Outcomes

The pre-specified primary outcome was refill adherence to statins based on pharmacy dispensing records. Refill adherence was assessed by calculating the proportion of days covered of the 360 days following the index date by dividing the total days' supply by the number of days of study participation [PDC360 (Hess et al., 2006)]. The index date is the date of the first prescription for a statin after selection by SFK. The total days supplied was calculated as the sum of days dispensed within the study period. If a supply exceeded the end of the study participation, this supply was corrected for exceeding the end of the period. The number of days of study participation was defined as the number of days between the index date and the index date + 360 or the last refill date, whichever came first. For assessing the last refill date, all refills for any drug were included. We analysed refill adherence both as a continuous measure and as a dichotomous measure, the latter with a threshold of 80%: patients with a PDC360 < 80% were defined as non-adherent and patients with a PDC360 ≥ 80% were defined as adherent.

The secondary outcome was the occurrence of complete discontinuation, defined as more than 182 consecutive days (50%) of the 1-year observation period uncovered.

### Power calculation

With a type one error (α) for a two-sided test of 0.05 and a probability of correctly rejecting the false null hypothesis of 0.80 (1−β) 69 patients were needed in each of the 3 arms to demonstrate an improvement in the proportion of adherent patients from 65 to 80% (Campbell et al., 1995). Assuming a dropout rate of 25% (Van Wijk et al., 2005), at least 269 patients were required. Each community pharmacist was asked to recruit at least 15 non-adherent patients, 5 patients in each group.

### Handling and storage of data and documents

All patient data were provided to the SFK by the participating pharmacies according to a pre-existing procedure to protect the study subjects' privacy. The SFK provided the data to the researchers at Utrecht University. All data with regard to the patients' identity were anonymized by the participating pharmacies.

### Intention to treat vs. per protocol

In daily practice, a healthcare provider can decide not to follow treatment guidelines or a study protocol. In this study, a pharmacist may have had good reasons not to invite a patient after randomization. Examples are no telephone number being known to the pharmacist or the patient having experienced a major life event like the death of a partner. In the counseling/ERD group, it was to be expected that a proportion of the patients would not be willing to visit the pharmacy for counseling. Since this could introduce a bias, we performed a per protocol (PP) analysis as well as an intention to treat (ITT) analysis. In the PP analysis, we only included the patients who received the intervention. In the ITT analysis, we included all randomized patients, even when a pharmacist decided for a specific patient not to follow the study protocol or when a patient was not willing to visit the pharmacy for counseling.

### Statistical analysis

The primary analysis was based on the ITT principle. Patient characteristics between groups were compared using Student's *t*-test or χ^2^-test. As the PDC360 was not normally distributed, we analysed the PDC360 between groups using the non-parametric Mann–Whitney U test (SPSS for Windows version 20.0). We used logistic multilevel analysis to study the effect on the dichotomous primary outcome (MLWIN for Windows version 2.22). The included levels were patient, GP and pharmacist. The secondary outcome of complete discontinuation was assessed using Cox proportional hazards. We considered a *p*-value of less than 0.05 to be statistically significant. In a second analysis, the following baseline values were considered as possible confounders and effect modifiers: age, gender, refill rate in 12 months prior inclusion, Chronic Disease Score (CDS), use of beta-blocking agents (BBA) or calcium channel blockers (CCB) and use of statin for secondary prevention. The CDS uses drugs dispensed as surrogate markers for chronic illness (Von Korff et al., 1992). Secondary prevention was defined as either concomitant use of one or more platelet aggregation inhibitors (PAIs, ATC code B01AC) and/or oral antidiabetic drugs (OAD, ATC code A10B). Effect modification was defined as a significant interaction (*p* < 0.10) between group allocation and the variable in question.

## Results

### Patient enrolment and baseline

A total of 399 patients considered eligible by the pharmacists were randomly assigned to one of the two intervention groups or the control group (Figure 1). Two patients were excluded because they did not fill any prescription after the selection date. A total of 16 patients were excluded because they started receiving medication weekly after the index date.

Patient characteristics and use of medication at baseline are presented in Table 1. Due to missing refill data before inclusion, it was impossible to compare the use of medication of 8 patients in the counseling/ERD group and 2 in the control group. Patient characteristics were similar but differences were found for the use of medication. More patients in the ERD group used BBAs than in the control group and fewer patients in the counseling/ERD group used CCBs than in the control group. The CDS within the three groups was comparable. There were no statistically significant differences in refill adherence before the index date (Mann–Whitney U test).

**Table 1:** **Baseline characteristics of study population**.

**Characteristic**	**Counseling with ERD (*n* = 130)**	**ERD only (*n* = 123)**	**Control group (*n* = 128)**	**Overall (*n* = 381)**
Age, mean [*SD*], years	73.3 [6.6]	73.2 [5.8]	73.9 [6.5]	76.5 [6.3]
Male, *n* (%)	61 (46.9)	53 (43.1)	54 (42.2)	168 (44.1)
Co-medication, *n* (%)	(*n* = 122)^*^	(*n* = 123)^*^	(*n* = 126)^*^	(*n* = 371)^*^
Oral antidiabetics (OAD)	26 (21.3)	26 (21.1)	32 (25.4)	84 (22.6)
Insulin without OAD	4 (4.2)	5 (5.2)	5 (5.3)	14 (4.9)
Thiazide diuretics	31 (25.4)	36 (29.3)	31 (24.6)	98 (26.4)
β blocking agents (BBA)	34 (35.2)	62 (50.4)	44 (34.9)	149 (40.2)
Calcium channel blockers (CCB)	11 (9.0)	25 (20.3)	27 (21.4)	63 (17.0)
Nitrates (sublingual)	10 (8.2)	19 (15.4)	12 (9.5)	41 (11.1)
Nitrates (oral, transdermal)	6 (4.9)	11 (8.9)	9 (7.1)	26 (7.0)
Antithrombotics	65 (53.3)	65 (52.8)	65 (51.6)	195 (52.6)
ACE inhibitors	31 (25.4)	32 (26.0)	41 (32.5)	104 (28.0)
Angiotensin II receptor blockers	22 (18.0)	28 (22.8)	25 (19.8)	75 (20.2)
Platelet aggregation inhibitor (PAI)	56 (45.9)	55 (44.7)	56 (44.4)	167 (45.0)
**STATIN, *n* (%)**				
Simvastatin	68 (55.7)	72 (58.5)	78 (61.9)	218 (58.8)
Pravastatin	9 (7.4)	10 (8.1)	7 (5.6)	26 (7.0)
Atorvastatin	26 (21.3)	28 (22.8)	29 (23.0)	83 (22.4)
Rosuvastatin	12 (9.8)	9 (7.3)	10 (7.9)	31 (8.4)
Fluvastatin	6 (4.9)	4 (3.3)	–	10 (2.7)
Simvastatin/ezetimb	1 (0.8)	–	2 (1.6)	3 (0.8)
Chronic Disease Score, mean [*SD*]	5.0 [2.4]	5.6 [3.1]	5.4 [2.8]	5.4 [2.8]
**REFILL RATE IN YEAR PRIOR TO INCLUSION**				
50–66%, *n* (%)	43 (35.2)	48 (39.0)	45 (35.7)	136 (36.7)
67–76%, *n* (%)	38 (31.1)	34 (27.6)	42 (33.3)	114 (30.7)
77–80%, *n* (%)	41 (33.6)	41 (33.3)	39 (31.0)	121 (32.6)

Note:

* *Missing refill data prior inclusion of eight patients in counseling/ERD group, 0 in the ERD group and 2 in control group*.

Baseline characteristics were also compared based on PP analysis, but this did not substantially change our findings (data not shown).

### Execution of the interventions

**Figure 3:**
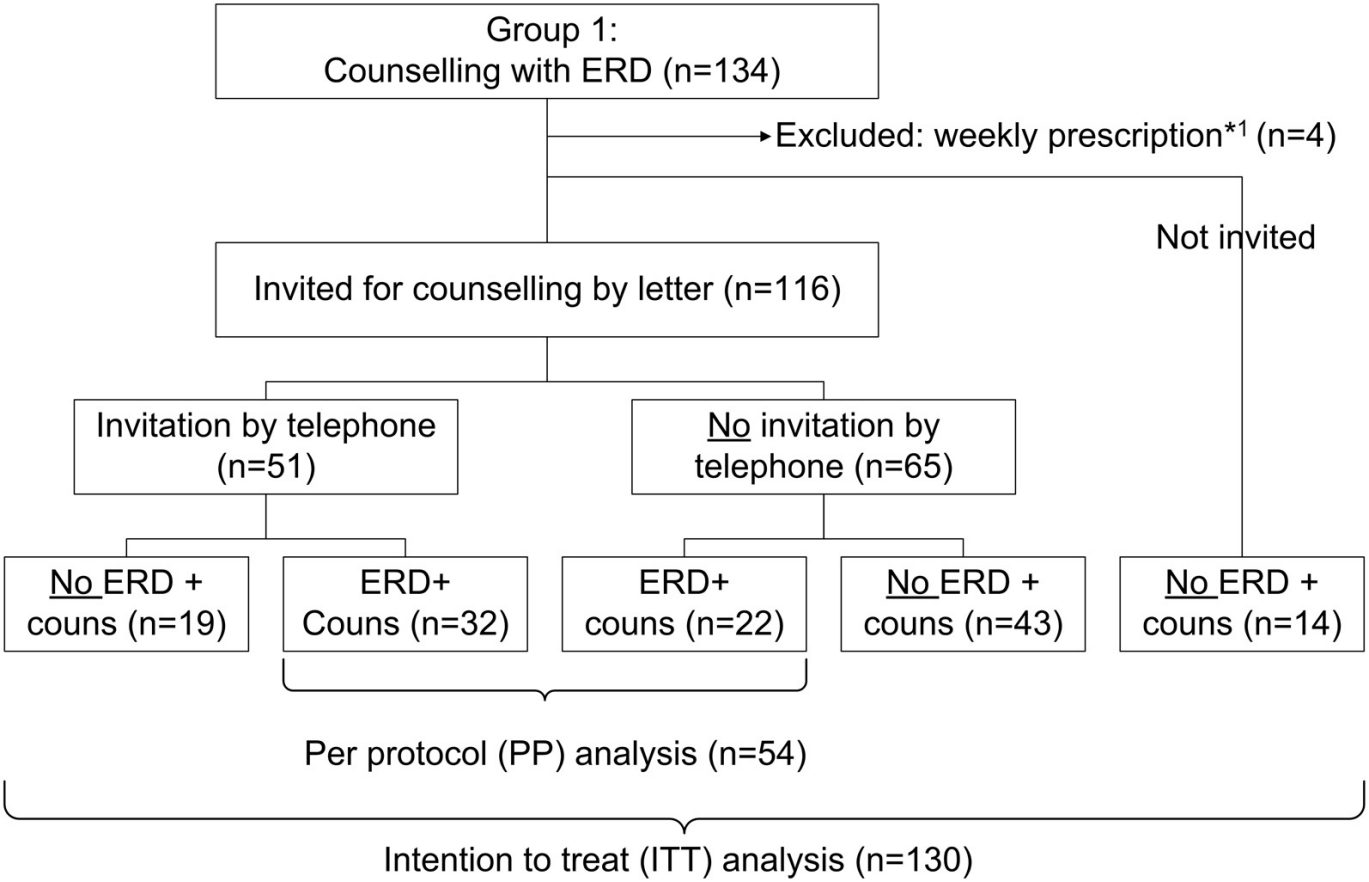
**Information about inclusion of patients in ERD/counseling group**.

Of the 134 eligible patients randomized to the counseling/ERD group, 4 patients became ineligible because they received their medication in week boxes, 116 patients were invited for counseling, and 14 patients were not invited for counseling (see Figure 3). Of the 14 patients not invited: 2 pharmacists did not register an invitation for any patient for counseling (*n* = 6), 1 pharmacist registered an invitation for 6 patients, and 1 pharmacist excluded 2 patients after randomization. Six pharmacists did not call any patient after the invitation by letter (*n* = 32). Sixteen pharmacists invited 51 out of 116 patients by phone as well and 32 (63%) of these patients actually received the counseling. Of the 65 patients who were not invited by phone as well, 22 (34%) patients received the counseling with ERD. In total 54 of the 116 invited patients (47%) eventually received counseling and the ERD.

Out of the 123 eligible patients in the ERD group 117 (95%) actually received the ERD. Two pharmacists did not send any patient the ERD (*n* = 6), but these pharmacists did invite patients for the counseling with ERD intervention.

### Primary outcome: refill adherence

The median PDC360 (25–75th percentile) was 90.0% (76.75–98.25) in the counseling/ERD group, 91.0% (76.00–99.00) in the ERD group and 87.5% (75.00–99.00) in the control group (ITT analysis). No statistically significant differences in the median refill adherence were assessed (Mann–Whitney U test). Using a cut-off of 80% (PDC360) 69.2% of the patients in the counseling/ERD group were adherent compared to 72.4% in ERD group and 64.8% in the control group (Table 2). These differences were not significant. Since the proportions are high, the presented odds ratios overestimate the relative risk and should therefore not be interpreted as such (Davies et al., 1998). A PP analysis revealed that 70.4% of the 54 patients in the counseling/ERD group were adherent and 72.6% of the 117 patients in the ERD group (Table 2). In a second analysis we assessed the effect of the intervention in different subgroups shown in Table 2. The use of OAD and/or PAIs was a significant effect modifier (*p* < 0.1). In patients not using OAD and/or PAIs there was no significant difference between the ERD group and the control group. Gender was only a significant effect modifier (*p* < 0.1) in the group of patients using OAD and/or PAIs. In the ERD group more women using OAD and/or PAIs were adherent (86.1%) compared to the control group (52.6%). This difference is statistically significant (*p* < 0.005). No effect of the ERD was found for men using OAD and/or PAIs.

**Table 2:** **Results of multilevel analyses of the effectiveness of the interventions on proportion of adherent patients (PDC360 > 80%)**.

**Study population**	**No. of subjects**	**No. of adherent subjects (%)**	**Crude model**		Adjusted model^*^	
			***OR* [95% CI]**	***p***	***OR* (95% CI)**	***p***
**INTERVENTION GROUP**						
Overall, intention to treat						
Control group	128	83 (64.8)	Ref.	NA	Ref.	NA
Counseling with ERD	130	90 (69.2)	1.22 [0.72–2.06]	0.45	1.18 [0.69–2.01]	0.55
ERD only	123	89 (72.4)	1.33 [0.76–2.32]	0.55	1.49 [0.83–2.69]	0.18
Overall, per protocol						
Control group	128	83 (64.8)	Ref.	NA	Ref.	NA
Counseling with ERD	54	38 (70.4)	1.29 [0.65–2.56]	0.47	1.25 [0.62–2.52]	0.54
ERD only	117	85 (72.6)	1.35 [0.77–2.36]	0.30	1.49 [0.83–2.68]	0.18
**SUBGROUP ANALYSIS (BASED ON INTENTION TO TREAT ANALYSIS)**						
Primary prevention						
Control group	52	37 (71.2)	Ref.	NA	Ref.	NA
ERD only	51	32 (62.7)	0.68 [0.29–1.57]	0.36	0.60 [0.24–1.48]	0.26
Secondary prevention, women						
Control group	38	20 (52.6)	Ref.	NA	Ref.	NA
ERD only	36	31 (86.1)	**5.58 [1.79–17.4]**	0.003	**8.26 [2.20–31.0]**	0.002
Secondary prevention, men						
Control group	38	26 (68.4)	Ref.	NA	Ref.	NA
ERD only	36	26 (72.2)	1.29 [0.46–3.67]	0.63	1.22 [0.36–4.11]	0.75

*Presented odds ratios are the ratios of proportion of adherent patients in intervention group vs. proportion in control group. When OR > 1: the odds of being adherent in the intervention group are higher than the odds in the control group*.

*Note: OR, odds ratio; CI, confidence interval; ERD, electronic reminder device*.

* *Adjusted model is corrected for refill adherence in 12 months before index date and use of beta-blocking agents (BBA) or calcium channel blocker (CCB)*.

*Significant associations (p < 0.05) are printed bold*.

### Secondary outcome: discontinuation

In the counseling/ERD group 6.2% (8) of the patients discontinued treatment with statins, compared to 5.7% (11) in the ERD group and 9.4% (12) in the control group. The adjusted hazard ratio for the counseling/ERD group vs. control group was 0.67 [95% CI 0.27–1.6] and for the ERD group 0.65 [0.25–1.7] (Table 3).

**Table 3:** **The effectiveness of the interventions on proportion of patients that discontinued therapy over time assessed using Cox proportional hazards**.

	**No. of subjects**	**No. of discontinued subjects (%)**	**Crude model**		Adjusted model^*^	
			***HR* [95% CI]**	***p***	***HR* [95% CI]**	***p***
**GROUP**						
Control group	128	12 (9.4)	Ref.	NA	Ref.	NA
Counseling with ERD	130	8 (6.2)	0.64 [0.26–1.6]	0.64	0.67 [0.27–1.6]	0.37
ERD only	123	7 (5.7)	0.60 [0.24–1.5]	0.29	0.65 [0.25–1.7]	0.37

Note:

* *Adjusted model is corrected for age at inclusion*.

The influence of our pre-specified threshold for optimal refill adherence was assessed in a sensitivity analysis and no influence on our primary conclusion was found (Table 4).

**Table 4:** **Result of sensitivity analysis: number and percentage of adherent patients when different thresholds were used for the definition of “adherent**.”

**Threshold**	**ERD with counseling (*n* = 130)**		**ERD only (*n* = 123)**		**Control group (*n* = 128)**
	***n* (%)**	***OR* [95% CI]**	***n* (%)**	***OR* [95% CI]**	***n* (%)**
PDC ≥ 75%	101 (77.7)	0.96 [0.52–1.77]	98 (79.7)	1.02 [0.92–1.13]	100 (78.1)
PDC ≥ 80%	90 (69.2)	1.18 [0.69–2.01]	89 (72.4)	1.49 [0.83–2.69]	83 (64.8)
PDC ≥ 85%	76 (58.5)	1.16 [0.68–1.98]	76 (61.8)	1.48 [0.84–2.59]	70 (54.7)
PDC ≥ 90%	66 (50.8)	1.26 [0.75–2.13]	66 (53.7)	1.60 [0.94–2.73]	66 (44.5)
PDC ≥ 95%	49 (37.7)	1.12 [0.67–1.90]	51 (41.5)	1.09 [0.96–1.23]	51 (34.4)

*Analysis based on intention to treat analysis*.

*Note: n, number of adherent subjects with the specified threshold. Based on multilevel analysis and corrected for refill adherence in 12 months before index date and use of beta-blocking agents (BBA) or calcium channel blocker (CCB)*.

## Discussion

### Main findings

In this effectiveness study we compared two interventions, (1) counseling with an ERD and (2) only an ERD, with usual care (control) and studied the effects on refill adherence and persistence. In the ITT analysis we found a small improvement in refill adherence in the overall population in both intervention groups, but this was not statistically significant. After stratification the effect of the ERD was particularly strong in female patients using statins for secondary prevention but not in men. Although this might be a chance finding, we believe there is an explanation for this result. Differences in adherence between groups of patients have been found (Jackevicius et al., 2002; Ye et al., 2007; Mann et al., 2010), Some recent studies show that women with coronary heart disease (Hammond et al., 2012) or MI (Carey et al., 2012; Kirchmayer et al., 2012), are less adherent to statins than men. However another study showed no difference between men and women after MI (Choudhry et al., 2008). Diabetes is an indication to prescribe statins for secondary prevention. Also in this group, women are less adherent than men (Yang et al., 2009) Gender differences exist in clinical management (Crilly et al., 2007; Fu et al., 2011). As far as we know, these gender differences in clinical management have not been studied in the Netherlands, but this might explain the lower adherence in women and consequently the larger effect of the intervention in women using statins for secondary prevention (Yang et al., 2003; Chapman et al., 2005; Perreault et al., 2005). In the control group we also found that women using statins for secondary prevention were less adherent (52.6%) than men (68.4%). So this might partly explain the positive effect in women.

The effects of reminder devices on refill adherence have been studied in populations, including patients with hypertension (Christensen et al., 2010) and patients using statins (Vrijens et al., 2006). Among patients with hypertension, use of an ERD improved adherence to an antihypertensive drug during a measurement period of 6 months. After 6 months the device had to be returned to compile the electronic monitoring data. There was a large dropout due to patients being unwilling to use the device, patients not returning the device or patients not providing self-reported adherence. This is different from our study where patients did not have to return the device or complete a questionnaire. In the study by Vrijens, the intervention was more complex and labor intensive than our intervention in the counseling/ERD group: at each follow-up visit the data of the electronically compiled dosing history were analysed together with the patient and the study also made use of a Medication Electronic Monitoring System. Their study found an improved adherence mainly by improving persistence. In our study we found no effect of the ERD on persistence (Table 3). Another difference between the two mentioned studies and our study is that we used refill data and not electronic monitoring data.

### Strengths of the study

Although the interventions showed no significant improvement in adherence in the overall study population, we showed that a very simple intervention of sending an ERD to non-adherent statin users can significantly improve medication refill adherence in women using statins for secondary prevention. Our study confirms the conclusion of Schedlbauer et al. (2010) that reminding patients appears to be the most promising intervention for improving adherence to statins. Many successful adherence-improving interventions are time consuming and labor intensive (Kripalani et al., 2007) and this hampers implementation in daily practice. Simple interventions that are easy to implement in daily practice for both the patient and healthcare professional offer the most promise for improving adherence (van Dulmen et al., 2008). But the challenge is to determine for which group of patients a simple intervention is effective and which group of patients need more tailored care. An example is the studied intervention with the ERD. This was easy to implement in daily practice and did not require much more than sending an instruction to the pharmacies and providing the pharmacies with the devices, letters for the patients and tools to select patients. So the ERD intervention was easier to implement in daily practice than counseling.

### Limitations of the study

Our study has some limitations. Firstly, some pharmacists did not follow the study protocol: they did not invite any patient for the counseling and they did not send the ERD with instructions. It would appear that some pharmacies did not follow the protocol completely, as they did not register an invitation for counseling, excluded after randomization or did not sent the ERD. Also some pharmacists did not invite the patient by telephone. For an individual patient there might be a good reason not to follow the protocol, for example, if the patient has recently experienced a life event and adherence is therefore not the most important issue at that moment in time. In the ITT analysis this diluted the effect of the intervention since all presented patients were included in the analysis. Only 54 of the 116 invited patients actually received the counseling. One important reason for this is that 65 patients did not receive a follow-up phone call from the pharmacist. Apparently, an invitation letter alone is not enough to motivate patients to visit the pharmacy. In the PP analysis we only included patients who eventually received the counseling or ERD. However, we believe that the effect of a selection bias is small, as pharmacists did not selectively exclude patients after randomization: they invited all or none. More attention should have been given to the implementation of the intervention with counseling. Secondly, the number of included patients is not quite high enough and so there might have been too little power to demonstrate a statically significant effect. Thirdly, some patients might have been selected as non-adherent, whereas in practice they were more than 80% adherent, for example when they were hospitalized. However, this is likely to be non-differentially distributed among our trials arms and so would only have diluted the effect of the intervention. Fourthly, in both intervention groups we do not know if the patients who received the ERD with the instruction actually used the device and neither do we know the patients' opinion. Future studies should investigate this in more detail. Finally, we used a multilevel logistic regression analysis that results in an odds ratio as an effect size. But since the proportions of adherent patients are relatively high, the odds ratio will overestimate the effect size when it is interpreted as a relative risk (Davies et al., 1998). So although we can make a statement as to whether there is an effect of the intervention, it is difficult to determine the actual size of the effect.

### Implications for practice and research

The results of this study suggest that the use of a simple ERD can improve refill adherence in specific subgroups of patients but not in the overall population. This justifies future studies aimed at more accurately quantifying the effect in different groups of patients. When an intervention for ERD use is designed we advise focusing on persistent, but non-adherent patients. Some of the patients in our study were not motivated to visit the pharmacy for counseling. This group requires attention and other types of counseling such as telephone counseling or home visits might be more appropriate for them.

## Conclusions

In this randomized controlled trial, we found no statistically significant improvement of refill adherence when an ERD was used with or without counseling. However, in a subgroup of women using statins for secondary prevention the ERD-improved adherence was statistically significant.

## Author contributions

We thank P. A. G. M. de Smet^2,3^, M. Teichert^3^, M. Westein^3,4^, E. Vogels-Giessen^5^ and O. H. Klungel^1^ for their work and input on the study design. We thank P. C. Souverein^1^ for his help in analysing the data. The results of this study were presented at the 15th Annual Meeting of Espacomp in Ghent, Belgium on Saturday 27 October 2012.

^1^ Department of Pharmacoepidemiology and Clinical Pharmacology, Utrecht Institute for Pharmaceutical Sciences, Utrecht University, Netherlands
^2^ Department of Clinical Pharmacy, Radboud University Nijmegen Medical Centre, Nijmegen, Netherlands
^3^ Royal Dutch Pharmacists Association (KNMP), The Hague, Netherlands
^4^ Federation of Patients and Consumer Organisations in Netherlands, Utrecht, Netherlands
^5^ Service Apotheek BV, Enter, Netherlands

M. Teichert, PharmD, PhD, epidemiologist, Royal Dutch Pharmacists Association (KNMP), The Hague, Netherlands

M. Westein, PharmD, Royal Dutch Pharmacists Association, The Hague, Netherlands and Federation of Patients and Consumer Organisations in Netherlands, Utrecht, Netherlands

O. H. Klungel, PharmD, PhD, associate professor of pharmacogenetics, Department of Pharmacoepidemiology and Clinical Pharmacology, Utrecht Institute for Pharmaceutical Sciences, Utrecht University, Netherlands

E. Giessen, PharmD, Service Apotheek BV, Enter, Netherlands

P. A. G. M. de Smet, PharmD, PhD, Professor of Pharmaceutical Care, Department of Clinical Pharmacy, Radboud University Nijmegen Medical Centre, Nijmegen, Netherlands, and Scientific Institute of Dutch Pharmacists, The Hague, Netherlands.

## Funding

This trial was funded by Utrecht University.

### Conflict of interest statement

The authors declare that the research was conducted in the absence of any commercial or financial relationships that could be construed as a potential conflict of interest.

## Appendix

### Appendix 1: translated invitation letter for counseling

Dear Sir/Madam,

In the Netherlands more than one million patients use cholesterol-lowering agents each year. As a user of … (simvastatin) you are one of them. These drugs must be taken every day. Research shows that many patients have difficulty adhering to this.

I would like to invite you to visit the pharmacy and talk about the use of … (simvastatin). Perhaps you have questions about this medicine, you may have experienced side effects or you might be uncertain about how to see it. We can discuss these things during our talk. As a pharmacist, I also need to know about how these drugs are used in practice and about any problems that might prevent their proper use. I would like to hear about your experience as a user.

Please could you be so kind as to contact the pharmacy to make an appointment. At the bottom of this letter you will find some suggestions for suitable dates and times. If none of these are convenient for you then please contact the pharmacy to see if another date and time is available.

Yours faithfully,

Your pharmacist

**Original text in Dutch:**

Geachte heer/mevrouw,

In Nederland gebruiken jaarlijks momenteel meer dan één miljoen patiënten cholesterolverlagende middelen. U als gebruiker van … (simvastatine) bent hier één van. Deze geneesmiddelen moeten iedere dag ingenomen worden. Uit onderzoek blijkt dat veel patiënten hier moeite mee hebben.

Graag zou ik u willen uitnodigen om langs te komen in de apotheek en te praten over het gebruik van … (simvastatine). Wellicht heeft u vragen over dit geneesmiddel, heeft u bijvoorbeeld last van bijwerkingen of twijfelt u aan de werking of het nut. Tijdens dit gesprek kunt u dit allemaal ter sprake brengen. Voor mij als apotheker is het ook belangrijk om te weten hoe dit soort geneesmiddelen in de praktijk gebruikt worden en of er problemen zijn die goed gebruik in de weg staan. Dat wil ik graag van u als gebruiker horen.

Onderaan deze brief vindt u een aantal tijdstippen waarvoor u een afspraak kunt maken door met uw apotheek te bellen. Mogelijk zit er geen tijdstip bij waarop u kunt. Na overleg met u kan er dan een ander tijdstip afgesproken worden.

Met vriendelijke groet,

Uw apotheker

### Appendix 2: translated information letter for ERD-only group

Dear Sir/Madam,

In the Netherlands more than one million patients use cholesterol-lowering agents each year. As a user of … (simvastatin) you are one of them. These drugs must be taken every day. Research shows that many patients have difficulty adhering to this.

To help you with your daily intake we are sending you a device. This device is designed specifically for people who need to take medicines every day. It gives a signal at a specific time. The next time you have to take … (simvastatin) just activate the device by pressing and holding this button, you will hear a beep. Afterwards, this device will warn you every day at the same time so that you remember to take … (simvastatin). You can then turn off the device by pressing the button. I hope this device helps you to take … (simvastatin) daily. You will not be charged for this advice.

If you have any questions about this letter then please do not hesitate to phone or to pop in to the pharmacy.

Yours faithfully,

Your pharmacist

**Original text in Dutch:**

Geachte heer/mevrouw,

In Nederland gebruiken jaarlijks momenteel meer dan één miljoen patiënten cholesterolverlagende middelen. U als gebruiker van … (simvastatine) bent hier één van. Deze geneesmiddelen moeten iedere dag ingenomen worden. Uit onderzoek blijkt dat veel patiënten hier moeite mee hebben.

Om u te helpen met de dagelijkse inname ontvangt u een apparaatje. Dit apparaatje is speciaal ontwikkeld voor mensen die dagelijks medicijnen moeten innemen. Dit apparaatje geeft op een door u ingesteld tijdstip een signaal af. De eerstvolgende keer dat u … (simvastatine) inneemt activeert u het apparaatje door de knop ingedrukt te houden, u hoort dan een piepsignaal. Vervolgens zal dit apparaatje u elke dag op hetzelfde tijdstip waarschuwen. Op deze manier wordt u er iedere dag aan herinnerd wanneer het tijd is … (simvastatine) weer in te nemen. U kunt het apparaatje dan uitzetten door nogmaals op de knop te drukken. Ik hoop dat dit apparaatje u helpt om … (simvastatine) dagelijks in te nemen. Er zijn voor u overigens geen kosten verbonden aan dit apparaatje.

Als u nog vragen heeft naar aanleiding van deze brief, dan kunt u natuurlijk altijd even bellen of langskomen.

Met vriendelijke groet,

Uw apotheker
